# Automaticity and Control in Prospective Memory: A Computational Model

**DOI:** 10.1371/journal.pone.0059852

**Published:** 2013-03-28

**Authors:** Sam J. Gilbert, Nicola Hadjipavlou, Matthieu Raoelison

**Affiliations:** Institute of Cognitive Neuroscience, University College London, London, United Kingdom; Ghent University, Belgium

## Abstract

Prospective memory (PM) refers to our ability to realize delayed intentions. In event-based PM paradigms, participants must act on an intention when they detect the occurrence of a pre-established cue. Some theorists propose that in such paradigms PM responding can only occur when participants deliberately initiate processes for monitoring their environment for appropriate cues. Others propose that perceptual processing of PM cues can directly trigger PM responding in the absence of strategic monitoring, at least under some circumstances. In order to address this debate, we present a computational model implementing the latter account, using a parallel distributed processing (interactive activation) framework. In this model PM responses can be triggered directly as a result of spreading activation from units representing perceptual inputs. PM responding can also be promoted by top-down monitoring for PM targets. The model fits a wide variety of empirical findings from PM paradigms, including the effect of maintaining PM intentions on ongoing response time and the intention superiority effect. The model also makes novel predictions concerning the effect of stimulus degradation on PM performance, the shape of response time distributions on ongoing and prospective memory trials, and the effects of instructing participants to make PM responses instead of ongoing responses or alongside them. These predictions were confirmed in two empirical experiments. We therefore suggest that PM should be considered to result from the interplay between bottom-up triggering of PM responses by perceptual input, and top-down monitoring for appropriate cues. We also show how the model can be extended to simulate encoding new intentions and subsequently deactivating them, and consider links between the model’s performance and results from neuroimaging.

## Introduction

In standard laboratory paradigms for assessing prospective memory (PM), participants are engaged in an ongoing task requiring classification of a series of stimuli. For example, on each trial the participant might be presented with a pair of letters, one upper-case and one lower-case, and respond with a left or right keypress to indicate the location of the upper-case letter [1]; [2]. In PM conditions, an additional instruction is introduced, for instance to press a middle button if the same letter is presented on both sides (e.g. “a A”). In this way PM targets are embedded within an ongoing task. However, these stimuli do not compel a PM response; participants could also make a standard ongoing response (in this case, right key) if they did not classify the stimulus as a target. In some respects this is similar to real-world situations in which people hold a delayed intention (e.g. to post a letter), while performing an ongoing task (e.g. walking down the street, engaged in conversation). On encountering a target (i.e. mailbox), one might make an appropriate PM response (post the letter), or miss the target and continue the ongoing task (walk past the mailbox).

Within the PM literature, a debate has arisen over the mechanisms by which PM responses are triggered. According to some authors [3], detecting a PM target is contingent upon the engagement of preparatory attentional processes, i.e. resource demanding processes that lead to appropriate monitoring of the environment for PM cues. Without such processes, it is argued, PM cues cannot be detected as such. Consistent with this account, several studies have shown that response times (RTs) in the ongoing task to nontargets are slowed when participants hold in mind a delayed intention [3]; [4]. This slowing (“PM task interference effect”) is taken to reflect the withdrawal of resources from ongoing task performance in order to permit monitoring for PM targets. Furthermore, at least in some studies, the size of each participant’s PM interference effect is correlated with the percentage of PM targets detected, suggesting that the PM interference effect is functionally related to detection of PM targets [3]. However, this relationship between the PM interference effect and PM target detection has not always been observed [5].

Additional evidence that could be taken to support monitoring theories comes from analyses of RTs on PM miss trials (where an ongoing response is made to a PM target) versus ongoing trials (where an ongoing response is made to a PM nontarget). It has been reported that (erroneous) ongoing responses made to PM targets have faster RTs than (correct) ongoing responses made to nontargets [6]. This can be considered an example of an ‘intention superiority effect’ [7], seeing as responses on trials associated with a delayed intention are speeded relative to nontarget trials (see [8] for further discussion). This pattern of results could be caused, at least in part, by a failure of preparatory monitoring on PM miss trials. This could lead to faster RTs than standard ongoing trials, on which preparatory monitoring will be engaged at least on a proportion of trials. In support of this hypothesis, West et al. [9] found that RTs on nontarget ongoing trials preceding a PM miss were faster than trials preceding a PM hit, suggesting that a disruption of preparatory monitoring, associated with faster ongoing RTs, predicted subsequent PM misses.

In contrast with monitoring theories, some authors have suggested that in certain circumstances PM cue detection can be triggered relatively automatically by presentation of the appropriate stimulus, in the absence of deliberate preparatory monitoring [4]; [10]; [11]; [12]. According to these accounts, deliberate target monitoring can still play a part in prospective remembering, but it need not be mandatory. For example, according to the multiprocess framework of McDaniel and Einstein [4]; [13], some situations encourage automatic detection of PM targets, whereas other situations require deliberate monitoring. According to McDaniel and Einstein [13], “focal” cues, in which the stimulus attributes defining PM and ongoing responses overlap, can sometimes lead to automatic PM target detection. By contrast, “nonfocal” cues, where PM targets and ongoing responses are related to different aspects of stimuli, or even different stimuli, are proposed to require monitoring. Evidence for multiprocess accounts comes from studies showing that the size of the PM interference effect is modulated by the nature of the PM and ongoing tasks, suggesting that monitoring is required to a greater or lesser degree depending on the nature of the task [4]; [14]. Furthermore, in some studies, accurate PM responding has been reported [11], or PM cues have been noticed [10], in the absence of a detectable PM interference effect (see also [15]; [16]).

Here, we attempt to address this debate by presenting a computational model simulating performance in a PM paradigm. The model is related to the earlier model of Gilbert and Shallice [17], which was itself an extension of the model presented by Cohen, Dunbar, and McClelland [18]. Gilbert and Shallice’s model simulated performance in a task switching paradigm, in which participants switch rapidly between different tasks on a trial-by-trial basis. The present model is conceptually related to this earlier work, seeing as PM paradigms require participants to switch from an ongoing to a PM response on target trials. Like the Gilbert and Shallice model, the present model is based on the Parallel Distributed Processing (PDP) framework, using the interactive activation equations introduced by McClelland and Rumelhart [19]. The model consists of several processing units (“nodes”), each with an associated activation value. Activation spreads between nodes, dependent on the weights of connections between them. A simulated trial begins with activation being applied to input units. Processing is then iterated in cycles, with activity propagating through the network on each cycle, gradually accruing in units to which the input units are connected. The trial ends when a threshold is met at the response units, determining that a particular response has been selected; the number of cycles taken to reach the response threshold is recorded as the model’s RT. Behaviour and RT can then be compared against analogous empirical results.

In the Gilbert and Shallice model, two possible tasks (colour-naming and word-reading in response to Stroop colour-word stimuli) were implemented as distinct input-output pathways, connecting input and output nodes. Empirical phenomena associated with task switching were simulated as a consequence of competition between these two pathways. A pair of ‘task demand’ units implemented top-down control, biasing processing towards one or the other pathway. On trials where one pathway was much stronger than the other, the model produced relatively fast responses. But on trials where the two pathways were more similar in strength (e.g. immediately after a switch of tasks), RT was extended as a result of competition between conflicting responses. The present model implements a similar mechanism. There are two pathways leading from input to output: one representing the ongoing task and one representing the PM task (i.e. detecting PM cues and pressing the PM response button). These input-output pathways represent relatively automatic responses triggered directly by perceptual input. In addition, a ‘monitoring unit’ implements top-down control, by selectively boosting activation along the PM pathway. Thus, competition between ongoing and PM responses is modulated by activation of the monitoring unit. Consistent with the multiprocess framework [13], PM responding is therefore triggered by a combination of mechanisms: direct triggering of the PM response by an automatic stimulus-response link, and top-down monitoring, which assists this PM stimulus-response link. Crucially, monitoring in the model is a graded phenomenon: the monitoring unit can be set to variable levels of activation or to zero, in which case it has no impact on processing at all. In this respect, the model differs from the theoretical account put forward by Smith and Bayen [20], which was implemented in a mathematical model whereby monitoring either takes place or does not on a particular trial, in an all-or-nothing fashion.

## The Model

### Task

The task simulated by the model was as described in the Introduction, and used empirically in the studies of Gilbert et al. [1] and Okuda et al. [2]. Possible inputs consist of a pair of letters from the set (A, B, C), with one letter presented in upper-case and one letter presented in lower-case. The appropriate response is left if the upper-case letter is on the left, and right if the upper-case letter is on the right. This constitutes the ongoing task. In addition, if the same letter is presented on both sides (e.g. “a A”), the appropriate response is to press the middle button rather than the right button. This constitutes the PM task.

### Model Architecture

**Figure 1:**
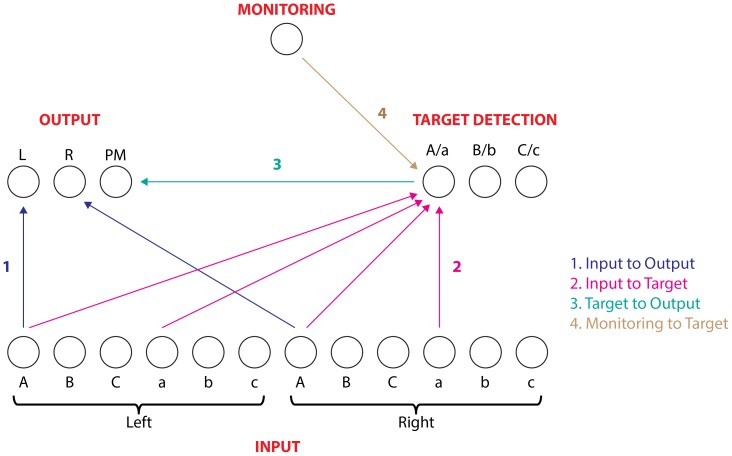
Model architecture. Only connections between units representing the letter ‘A’ are shown, for simplicity; analogous connections existed for representations of ‘B’ and ‘C’.

The architecture of the model is presented in Figure 1. There are 12 input units, representing each of the six possible stimuli at each position. For example, the stimulus “A c” would be simulated by activating the leftmost and rightmost input unit. The three output units represent Left, Right, and PM responses. The three input units representing an upper-case letter on the left are connected with positive connection weights to the Left output unit; likewise the units representing upper-case letters on the right send a positive input to the Right output unit. These connections (labelled 1 in Figure 1) constitute the direct stimulus-response pathway underlying the ongoing task. The PM input-output pathway involves an intervening set of ‘target detection’ units; thus the pathway is constituted by the connections labelled 2 and 3 in Figure 1. Each of the four input units representing the letter ‘A’ or ‘a’ is connected to the ‘A/a’ target detection unit; likewise for the units representing B/b and C/c. The three target detection units are all connected with a positive weight to the PM output unit. Thus, when a PM target stimulus is presented, both input units will send activation to the relevant target detection unit, which itself sends activation to the PM output unit. However, a nontarget stimulus will send activation to different target detection units, so no single unit will achieve a high degree of activation. The monitoring unit sends activation to each of the three target detection units. This top-down pathway (labelled 4 in Figure 1) is assumed to represent strategic, deliberate monitoring for PM targets. Thus activation of target detection units arises from both direct bottom-up triggering from the stimulus input units, and top-down modulation from the monitoring unit. Insofar as bottom-up triggering from the input units is insufficient for PM responding, top-down input from the monitoring unit is additionally required, as proposed in the multiprocess framework [13]. In addition to the connections described above, each of the three output units send a negative connection to the other two output units; likewise the three target detection units are connected to each other in a similar manner. This implements a form of lateral inhibition, whereby activation in one of the output or target detection units tends to suppress activation in the other two units. This encourages the model to converge on activation of a single unit within these modules.

### Operation of the Model

The model parameters were set by hand in order to produce adequate performance of the task (see Table S1 for values). The model is therefore potentially open to the criticism that it could capture any pattern of behaviour and that its parameters have simply been set so that it reproduces known empirical results, without providing any theoretical constraints. In order to address this point, it is important that the model is able to generate novel predictions that can be tested empirically. Insofar as the model makes such predictions, and they are empirically validated, this indicates that the model goes beyond simply reproducing pre-existing empirical results.

The steps taken to simulate a trial are as follows (essentially following the procedure used in the model of Gilbert and Shallice [17]). All units are initialized to an activation level of zero at the beginning of the trial. A “cycle” then takes place as follows. Two input units have their activation level set to the input activation level (to represent the stimulus presented on that trial) and the monitoring unit is set to the monitoring activation level (to represent the PM top-down monitoring level for that trial; see Table S1 for parameter settings). For the remaining units, the net input is calculated by summing the activation level of every other unit, multiplied in each case by the relevant connection weight to the target unit (see Table S1 for connection weights). In addition, there is a negative bias term added to the net input for each target detection unit, so that net input to these units will always be negative unless counteracted by positive activation contributed from other units. Once the net inputs have all been calculated, the activation level for each unit is updated as follows:
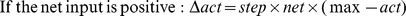


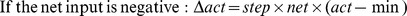



Where act = current activation, step = step size, net = net input, max = maximum activation value, and min = minimum activation value. The step size parameter determines the magnitude of the change in activation on each cycle, setting the speed of processing. On each cycle, a random noise term is also added to the activation values of every unit. This term is drawn from a Gaussian distribution, with a mean of zero; the standard deviation of this distribution determines how much disruption is caused by noise on each cycle. After noise has been added the activation levels of any units outside the maximum and minimum values are reset to the relevant extreme. At the end of each cycle, the activation level of the most active output unit is compared against the second most active output unit. If this difference exceeds a threshold, the response associated with the most active output unit is declared as the model’s response on that trial, and the number of cycles since the beginning of the trial recorded as the RT. Otherwise, the net inputs are calculated again and a new cycle begins. In this way, activation gradually propagates through the network until a response threshold is met. If the response threshold has not been met after 500 cycles, the trial is ended and an error recorded (without recording RT).

A fundamental feature of the model is interactive competition between the PM and ongoing pathways. On a nontarget trial, the relevant input unit (i.e. the unit representing the upper-case letter) will send activation to one of the ongoing response units, leading to a build-up of activation at the output layer. Seeing as nontarget stimuli do not lead to significant activation of the target detection units (because the two input units, sending activation to different target matching units, do not provide sufficient activation to counteract the negative bias applied to these units), the ongoing response unit will generally reach the response threshold, rather than the PM response unit. On a PM target trial, the two pathways will compete. Activation will be sent from the input units directly to the relevant ongoing output unit. In addition, activation will be sent from the input units, via the target matching units, to the PM response unit. Thus activation will build up in both the PM output unit and one of the ongoing output units. Due to the lateral inhibition between these units, the ongoing and PM output units will tend to inhibit each other. Thus, small differences in the relative input contributing to the ongoing and PM response units (including the noise added to the activation levels on each cycle) will have the effect of tipping the model’s output towards a PM or ongoing response, in a competitive manner.

### Performance of the Model

#### 1. Effect of monitoring level

**Figure 2:**
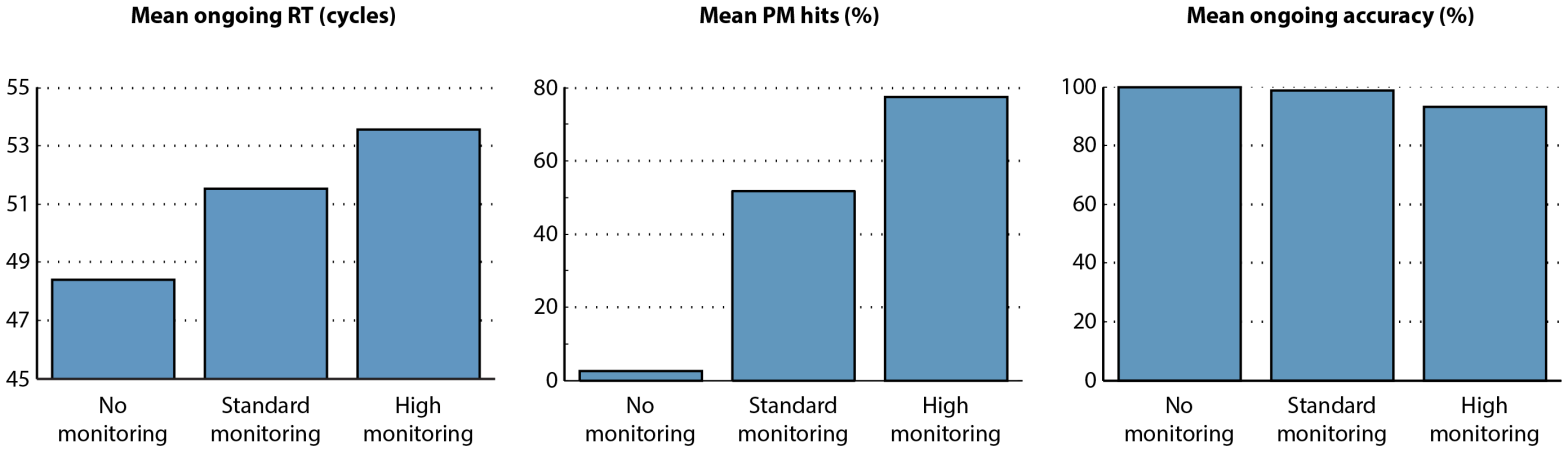
Model performance. Mean response time, PM hit rate, and ongoing accuracy in ‘No monitoring’, ‘Standard monitoring’, and ‘High monitoring’ settings.

In order to test performance of the model, 100,000 simulated trials were run of each of the 12 possible ongoing trials and 200,000 simulations of the six possible PM target trials, so that equal numbers of target and nontarget trials were run. This was using the standard parameter settings detailed in the Appendix. We refer to this as the ‘standard monitoring’ settings. Due to the large number of trials, all differences between conditions in the model’s performance were highly significant (generally p<10^−100^); we therefore omit significance testing in the results reported below. Two additional simulations were conducted, identical to the standard monitoring settings, but with the activation level of the monitoring unit set to 1 and zero. These settings are referred to as the ‘high monitoring’ and ‘no monitoring’ settings respectively. Results from these simulations are presented in Figure 2.

There are three noteworthy features of Figure 2. First, the model is able to perform the task adequately. Performance on nontarget ongoing trials is near ceiling, and the model is able to detect PM targets on at least a proportion of trials, even when monitoring is set to zero. Further analysis indicated that on PM miss trials, the correct ongoing response (e.g. Left rather than Right for ‘A a’) was produced on over 99% of trials, indicating that PM misses were associated with otherwise correct performance of the ongoing task. Second, higher top-down monitoring levels were associated with an increased proportion of hits on PM target trials. Third, higher top-down monitoring levels were associated with increased RT even on nontarget ongoing trials. Thus, the model simulates the intention maintenance cost, and the relation between this cost and accuracy of PM target detection, reported by Smith [3]. These findings are readily accounted for in computational terms, seeing as the monitoring unit sends direct input to the target matching units. On PM target trials, this will boost activation along the PM pathway, making it more likely that the PM output unit will win competition against the ongoing output unit. On nontarget trials, this boosting effect will lead to some activation being sent to the PM output unit, extending the response competition process between the ongoing and PM output unit and causing longer RTs.

#### 2. Effects of stimulus degradation

In a second analysis, we investigated whether any other factors, alongside top-down monitoring level, could affect the model’s PM target detection rate. In order to do this, we ran an additional simulation identical to the standard settings, except that the stimulus input level (i.e. activation level to which activated stimulus input units are set) was reduced from 1.0 to 0.9. This stimulates the effect of degraded stimulus input. We predicted that this manipulation would generally slow RTs, seeing as the contribution from the input units to other units in the model would be reduced, so it should take more cycles for the output units to reach the output threshold. We also predicted that this manipulation might affect PM target detection levels. Our reasoning was as follows. The pathway leading directly from input to output units (commanding ongoing responses) is direct. But the pathway leading from input to output units via target detection units (commanding stimulus-evoked PM responses) is indirect (i.e. involves intervening target detection units). It is therefore possible that degrading the input representations will have more effect on the indirect PM pathway (with an intervening set of units, and therefore an additional locus at which noise is added) than the direct ongoing pathway, which may be more robust to noise. As a result, competition between the ongoing and PM pathways will be biased somewhat towards the ongoing pathway, as a result of stimulus degradation.

**Figure 3:**
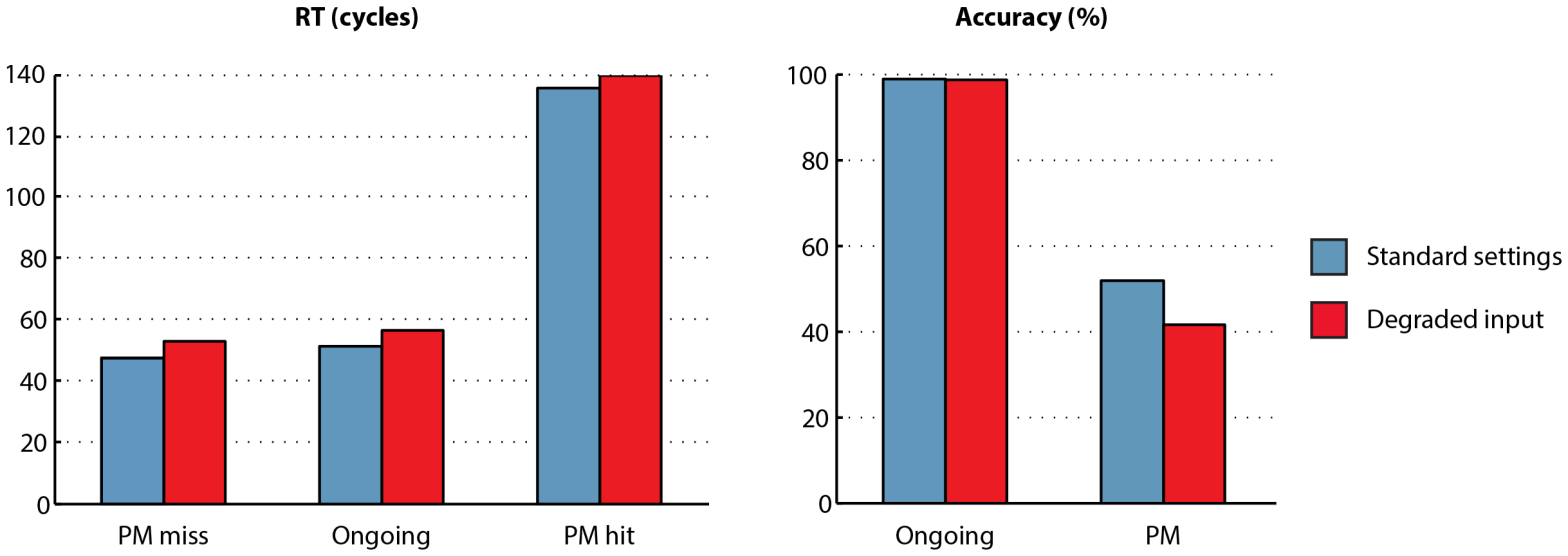
Effects of stimulus degradation. Mean response time for PM miss, correct ongoing, and PM hit trials, alongside accuracy for ongoing trials and PM hit rate. Results are shown separately for the model using its standard settings (blue bars) and degraded input settings (red bars).

Figure 3 shows that both of these predictions were substantiated. Stimulus degradation led to both increased ongoing RTs and decreased PM target detection. Thus, the model is able to capture a positive relationship between ongoing RT and PM target detection under some circumstances (manipulation of top-down monitoring) and a negative relationship between ongoing RT and PM target detection under others (degradation of stimulus input). The model’s simulation of the intention maintenance cost, i.e. the difference in ongoing RT between no-monitoring and standard-monitoring settings, was comparable in the standard settings (mean: 3.1 cycles) and the degraded input settings (mean: 3.4 cycles). The model’s behaviour is therefore compatible with empirical data showing a positive relationship between the intention maintenance cost and PM detection in some circumstances (e.g. [3]) but not others [5].

#### 3. Intention superiority effect

Inspection of Figure 3 shows that PM miss trials had faster RTs than ongoing trials, even though the model produces the same (i.e. ongoing) response for both. The model therefore simulates the intention superiority effect [6]. How can this be explained? The literature on this effect posits two potential explanations. The first is that stimuli associated with an active intention are represented at a higher level of activation than other types of stimuli [7]; [21], or in conjunction with distinctive motoric information [22], leading to a speeding of RT when that stimulus is encountered. This cannot explain the model’s simulation of the intention superiority effect, seeing as PM target stimuli are represented in an identical manner to other stimuli. PM target stimuli do lead to greater activation in target matching units than nontargets; however, the effect of this activity in the target matching units is to interfere with representations of left or right ongoing responses, not to aid them.

An alternative explanation is that the intention superiority effect reflects trials where top-down monitoring is absent or reduced, leading to faster ongoing RTs than nontarget trials, a proportion of which will be slowed by monitoring for PM targets [9]. Again, this explanation cannot explain the model’s performance, seeing as the monitoring unit was set to the same level on each time. Although there was slight fluctuation of the monitoring unit’s activation level on each cycle due to random noise, this had a trivial effect on the model’s performance and the intention superiority effect remained similar even when this noise was removed. How else might the intention superiority effect be explained? A clue comes from analysis of RT distributions, rather than simply examining mean RT.

#### 4. Response time distributions

**Figure 4:**
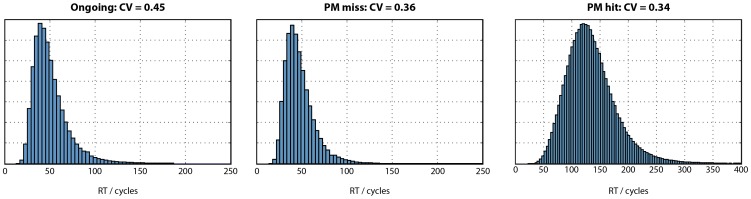
Response time distributions for the model’s simulation of correct ongoing, PM miss, and PM hit trials. Coefficient of variation (CV), i.e. standard deviation divided by mean, is also shown.

An important characteristic of the model is that noise is added to the activation level of each unit on each cycle. This gives rise to variability from one trial to the next, even when the stimulus is identical. We can therefore plot the frequency distribution of ongoing, PM miss, and PM hit RTs in Figure 4. Inspection of this figure suggests that these distributions are positively skewed, as commonly observed for RTs [23]. However, the right tail of the ongoing distribution seems slightly overrepresented, compared with the PM miss distribution (e.g. compare the small number of trials with RTs greater than 150 cycles in the ongoing distribution, versus the absence of such trials in the PM miss distribution). What could explain the “missing” right tail of the PM miss distribution? We propose the following explanation. As a result of random noise, on some trials the ongoing pathway will be favoured, relative to the PM pathway; on other trials the reverse will occur. Consider a trial on which noise particularly slows down the build up of activation in the relevant ongoing output unit. Of course, such trials will be associated with relatively slow ongoing responses, seeing as it will take many cycles until the activation level in the appropriate output unit reaches the response threshold. These trials will therefore comprise the right tail of the ongoing RT distribution. If a PM target has been presented, it is quite likely that sufficient activation will have accrued in the PM response unit to produce a PM response, before sufficient activation in the (slowly accumulating) ongoing response unit has accumulated for an ongoing response. This would therefore be counted as a PM hit, and the trial would not be included in the PM miss distribution. Conversely, those trials in which activation builds up particularly quickly in the relevant ongoing output unit will be likely to reach the response threshold before sufficient activation has accumulated in the PM response unit, and are therefore more likely to be included in the PM miss distribution. In other words, those trials in which activation builds up particularly quickly in the relevant ongoing response unit will be likely to be included in both the ongoing and PM miss distributions (i.e. when a nontarget and target stimulus are presented, respectively). These trials will have relatively fast RTs. However, those trials in which activation builds up slowly in the relevant ongoing response unit (due to noise) will be likely to be included in the ongoing distribution, but not the PM miss distribution (because they will be more likely to receive a PM hit). Thus, simply due to the effect of noise on the likelihood of a PM hit or miss, trials with slow RTs are unlikely to make it into the PM miss distribution, leading to a difference in mean RT between ongoing and PM miss trials. Of course, this explanation need not rule out additional explanations of the intention superiority effect, such as those discussed above. It does however suggest that an intention superiority effect could arise simply due to the effect of noise on competition between PM and ongoing response pathways.

One prediction of this account would be that a similar difference in the spread of the RT distributions should be seen when comparing ongoing trials with PM hits. On PM target trials where (due to noise) the PM response unit builds up activation quickly, a PM hit response is likely to be made. But on trials where activation in the PM response unit builds up slowly, an erroneous ongoing (i.e. PM miss) response is more likely to be produced. Thus, instead of being included in the right tail of the PM hit distribution, these trials may instead be included in the PM miss distribution. We would therefore expect that the PM hit distribution should have a less prominent right tail than the ongoing distribution (where trials in which evidence accumulates slowly will still, eventually, make it into the right tail of the distribution). Inspection of the PM hit distribution in Figure 4 shows this to be the case.

In order to quantify the spread of the ongoing, PM miss, and PM hit distributions, the coefficient of variation (CV) was calculated. CV is defined as the ratio of standard deviation to mean; it can therefore be considered to be a measure of variance, controlling for differences in mean RT between conditions [24]; [25]. Thus, proportional slowing of a RT distribution will lead to an increase in mean RT but no change in CV. In the model’s simulated RT distributions, CV was ordered ongoing >PM miss >PM hit.

We carried out additional analyses of the intention superiority effect in the model’s no-monitoring and high-monitoring settings. In the high-monitoring setting, the intention superiority effect was enhanced (9.8 cycles, or 18.2% of mean ongoing RT; standard settings: 3.8 cycles, 7.5% of mean ongoing RT) whereas in the no-monitoring setting the intention superiority effect was in fact slightly reversed (−0.8 cycles, 1.6% of mean ongoing RT). This suggests that in the no-monitoring settings, when PM hits were rare (3%), the additional response competition on PM target trials may have slowed ongoing responses, whereas in the conditions where PM hits were more common the effects described above played a greater role in determining the intention superiority effect. Evidence for response competition on PM target trials comes from paradigms where previously-relevant target stimuli cause slowing of RT on subsequent trials [26]; [27]. The simulations therefore suggest that under some conditions the intention superiority effect may not be obtained, and also that at least in some circumstances (e.g. when variability in PM hit rates is determined by monitoring levels) the size of the intention superiority effect should increase when PM hits are more common.

## Experiment 1

We have now seen that the model reproduces several empirical findings reported in the PM literature. It is able to perform the task adequately. Ongoing RT slows when the model is monitoring for PM targets, and the PM interference effect can be correlated with PM detection rate in some circumstances but not others. The model has also made two novel predictions. First, the model predicts that PM hit rate might be decreased, and ongoing RT increased, when stimuli are degraded. Second, the model predicts that ongoing RT distributions should have a greater coefficient of variation than either PM hit or PM miss distributions. We therefore conducted an empirical study to test these predictions.

### Methods

This research was approved by the UCL Division of Psychology and Language Sciences ethics committee. All participants provided written informed consent before taking part. 26 participants (17 female) took part in the study, in return for £5 or course credit. Their mean age was 25 years (standard deviation: 6.5). Two participants did not respond to any PM targets (making it unclear whether they had understood the task instructions) and a third made PM responses to fewer than 4% of targets, too few for an analysis of PM hit RT distributions. These three participants were excluded, alongside one further participant who fell asleep during the experiment, leading to a final sample of 22 participants.

**Figure 5:**
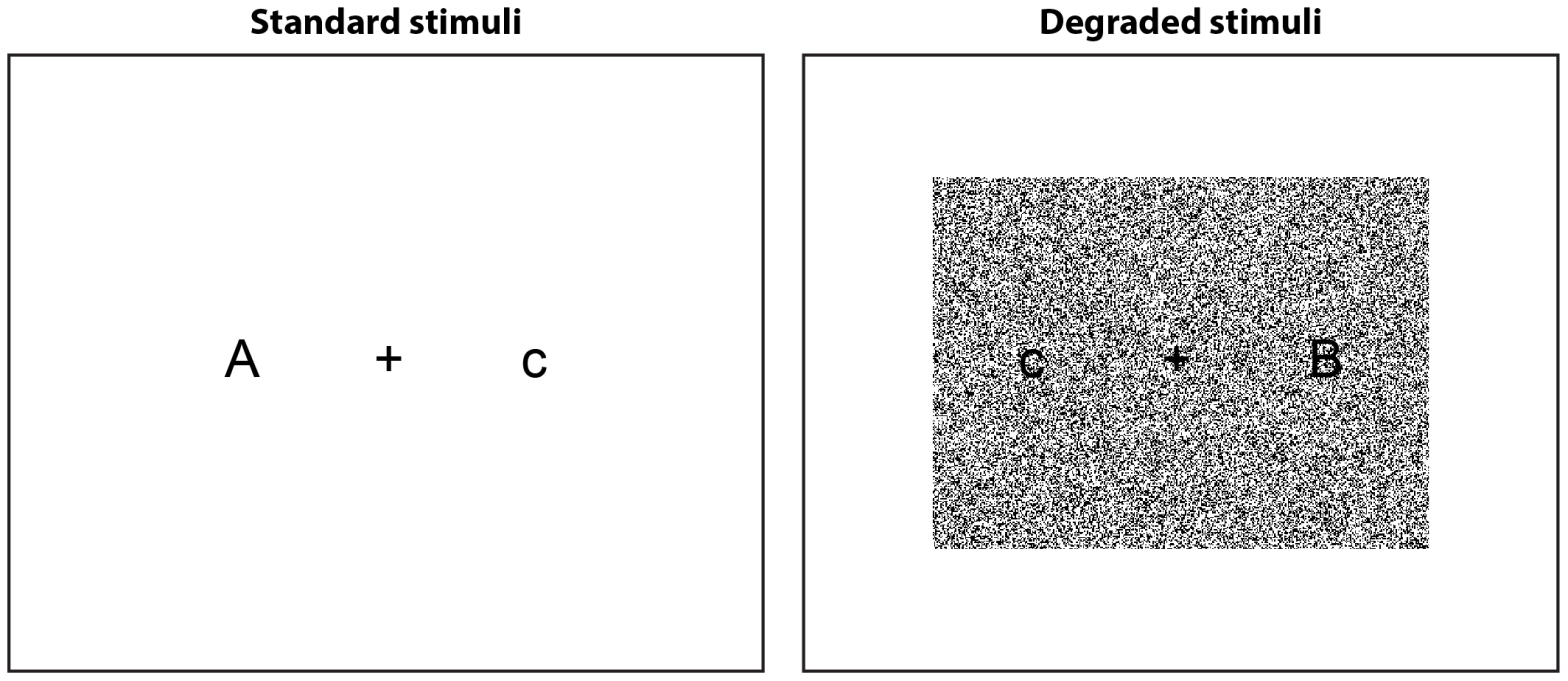
Schematic illustration of experimental stimuli in standard input and degraded input conditions.

Participants were tested individually, sitting approximately 50 cm from a laptop computer in a quiet testing room. Example stimuli are presented in Figure 5. Pairs of letters (A, B, or C, one upper-case and one lower-case) were presented in white Arial font (size 60) on a black background, with a fixation cross in the centre of the screen. In half of the blocks, stimuli were degraded by placing 85000 white pixels in randomly selected positions over a 400 by 300-pixel rectangle in the centre of the display (i.e. 71% of pixels in this rectangle). At the beginning of the experiment, the ongoing task was described to participants and they performed two blocks of 100 trials to familiarize themselves with the task (with standard stimuli). Participants made their responses using the leftmost and rightmost of three adjacent keys on the keyboard. The PM instructions were then explained: participants were instructed to press the middle button if they noticed that the same letter was presented on both sides of the screen. Without any further practice, the experiment then began. Participants performed 10 blocks of 100 trials, alternating between standard and degraded stimuli for each block (with the stimuli for the first block counterbalanced between participants). PM targets were presented on a randomly selected 8% of trials. On each trial, the stimulus was presented and remained on screen until a button was pressed. The screen was then blanked for a variable delay (100–300 ms) after which the next stimulus was presented.

## Results and Discussion

**Figure 6:**
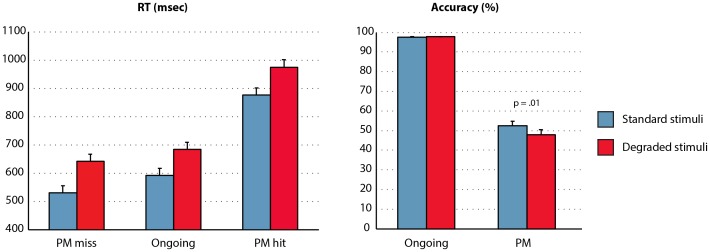
Empirical data. Mean response times are shown for PM miss, correct ongoing, and PM hit trials, alongside accuracy for ongoing trials and PM hit rate. Results are shown separately for the standard stimulus condition (blue bars) and degraded stimulus condition (red bars). Error bars indicate 95% confidence intervals for the within-subjects comparison between standard stimulus and degraded stimulus conditions, using Loftus and Masson’s [49] method. See Fig. 3 for equivalent data from the model.

The first trial of each block was excluded from the analysis. Only correct ongoing trials are included in the analysis of ongoing RTs (as in the model simulations above). Mean RTs and accuracy levels (i.e. proportion correct for ongoing trials and proportion of hits for PM target trials) are shown in Figure 6. RTs were analysed in a 3×2 ANOVA with factors Trialtype (PM miss, ongoing, PM hit) and Noise (noise, no-noise). There were significant main effects of Trialtype (F(2,20) = 85, p<.001, η^2^_p = _.90) and Noise (F(1,21) = 101, p<.001, η^2^_p_ = .83), but no significant Trialtype x Noise interaction (F(2,20) = 1.47, p = .25). Follow-up tests showed that PM miss trials had significantly faster RTs than ongoing trials (F(1,21) = 53, p<.001, η^2^_p_ = .72), and PM hit trials had significantly slower RTs than ongoing trials (F(1,21) = 90, p<.001, η^2^_p_ = .81). Furthermore, analysis of accuracy indicated that there were fewer PM hits in the noise than the no-noise condition (48.0% vs 52.4%; F(1,21) = 7.1, p = .01, η^2^_p = _.25). There was also a trend towards higher ongoing accuracy in the noise than the no-noise condition (97.8% vs. 97.5%; F(1, 21) = 3.9, p = .06, η^2^_p = _.16). Analysis of individual differences in PM hit rates showed that participants with higher hit rates also tended to have a larger intention superiority effect (r = .62, p = .002). Thus, the behavioural results were consistent with the following features of the model’s performance: 1) slower RTs for noise than no-noise conditions; 2) faster RT for PM miss than ongoing (i.e. intention superiority effect); 3) slower RT for PM hit than ongoing; 4) lower PM hit rate for noise than no-noise conditions; 5) greater intention superiority effect associated with increased PM hit rate. We are not proposing that degrading stimuli will always lead to a decrease in PM target detection, across all event-related PM paradigms. For example, in some situations, degrading stimuli might cause the feature distinguishing targets versus nontargets to become more salient. However, the present results indicate a situation where a single manipulation can lead to both slowed ongoing RTs and decreased PM target detection, consistent with the model’s performance.

### 

#### RT distributions

**Figure 7:**
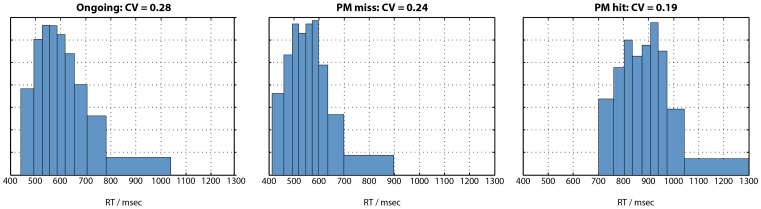
Response time distributions for correct ongoing, PM miss, and PM hit trials. Distributions have been averaged over participants using Ratcliff’s [28] method, with 10 bins. Coefficient of variation (CV), i.e. standard deviation divided by mean, is also shown. As in the model’s simulations (Fig. 4), CV is greatest for ongoing trials, intermediate for PM miss trials, and least for PM hit trials.

We next examined the model’s predictions concerning RT distributions. Figure 7 illustrates the average RT distribution for ongoing, PM miss, and PM hit trials. These data were generated using Ratcliff’s [28] method for generating group RT distributions, using 10 bins and collapsing over noise and no-noise conditions to maximize power. As in the model’s stimulations, the ongoing condition is least symmetrical of the three, with a long right tail. Coefficient of variation (CV) was calculated for the ongoing, PM miss, and PM hit distributions to test the model’s prediction of greater CV for ongoing trials than PM hit trials, with an intermediate CV for PM miss trials. The same pattern was seen in the empirical data (Figure 7), albeit with lower CV than the model across all conditions. CV was compared between conditions using Wilcoxon signed rank tests (due to significant deviation from the normal distribution). CV for ongoing trials was significantly greater than for PM hit trials (p<.001) and also PM miss trials (p = .0495). However, it should be noted that by virtue of PM trials being relatively rare, ongoing and PM trials had very different sample sizes. Seeing as CV may be influenced by sample size, an unbiased estimate of CV for the ongoing condition was calculated as follows. For each participant, a random sample of trials from the ongoing condition was obtained, equal to the number of PM hit trials. The CV for this ongoing distribution, matched in sample size to the PM hit distribution, was then calculated. This procedure was repeated 100,000 times to obtain a mean unbiased CV for the ongoing condition. An analogous procedure was used to calculate an unbiased ongoing CV for comparison against the PM miss distribution. Again, in these analyses CV was significantly greater for ongoing than PM hit trials (p<.001) and marginally significantly greater for ongoing than PM miss trials (p = .077). Thus, the model’s predictions were confirmed. Compared with ongoing trials, CV was reduced on both PM hit trials (associated with significantly slower mean RT) and PM miss trials (associated with significantly faster mean RT). Finally, the unbiased analyses were repeated, separately for the standard and degraded stimuli. With standard stimuli, the CV for ongoing trials (.24) was significantly greater than the CV for PM hits (.18; p = .006) and PM misses (.20; p = .016). With degraded stimuli, the CV for ongoing trials (.24) was significantly greater than the CV for PM hits (.17; p<.001). The comparison against the CV for PM misses (.23) was not significant (p = .10), although the trend was in the predicted direction.

## Experiment 2

A central feature of the model’s architecture is competition between the ongoing and PM input-output pathways so that on each trial either an ongoing or a PM response is made, but not both. The competitive interactions between these two pathways underlie the model’s novel predictions concerning response time distributions across different experimental conditions, as well as phenomena such as the intention superiority effect. Experiment 1, like many empirical studies of PM, also used a task in which participants were instructed to make either an ongoing or PM response on each trial, but not both. However, other studies have used a task in which participants are instructed to make PM responses in addition to ongoing responses on each trial, rather than instead of them (see [29] for discussion). To simulate this situation, the model would need to be modified. One simple way of doing this would be to make the ongoing and PM input-output pathways entirely separate, without lateral inhibition between response units and separate response thresholds for the two types of response. In this case, the model’s explanations of the intention superiority effect and differential response time distributions between ongoing, PM miss, and PM hit trials would no longer apply. The purpose of this experiment was therefore to directly compare these two experimental paradigms. After [29] we refer to these as the “task switching” condition (where participants should make an ongoing or a PM response, but not both) and the “dual task” condition (where PM responses should be made in addition to ongoing responses). We investigated whether these experimental paradigms would modulate the following three effects that, in the model, are dependent on competitive interactions between ongoing and PM pathways: 1) intention superiority effect; 2) greater CV of ongoing than PM miss RT distribution; 3) greater CV of ongoing than PM hit RT distribution.

### Methods

This research was approved by the UCL Division of Psychology and Language Sciences ethics committee. All participants provided written informed consent before taking part. 44 participants were recruited to take part in the study (26 female; mean age: 28, SD: 8). Two participants failed to make any PM responses, making it unclear whether they had understood task instructions, and one participant performed at chance level (48%) on the ongoing task. These three participants were excluded, along with an additional participant whose data were excluded due to technical problems, leaving a final sample of 40 participants. These participants were divided randomly into equally-sized task switching and dual task groups.

Participants were tested individually, sitting approximately 50 cm from a laptop computer in a quiet testing room. The task and procedure were identical to Experiment 1 with the following exceptions. First, stimuli were never visually degraded in Experiment 2. Second, stimuli were always presented for a fixed duration of 650 ms, after which a blank screen was presented for a random response-stimulus interval between 200–400 ms. Thus, stimulus presentation was identical between the two participant groups rather than being affected by responses produced on each trial. Participants first performed 75 practice trials of the ongoing task alone. The PM instructions were then explained. Participants in the task switching group were instructed to make a PM response instead of an ongoing response if they detected a target. Participants in the dual task group were instructed to make an ongoing response on every trial and to make an additional PM response if they detected a target. They were told that they could make the two responses in either order, but that they had to press the PM button before the onset of the next trial. After a further 75 practice trials, participants performed 10 blocks of 225 experimental trials.

## Results

**Figure 8:**
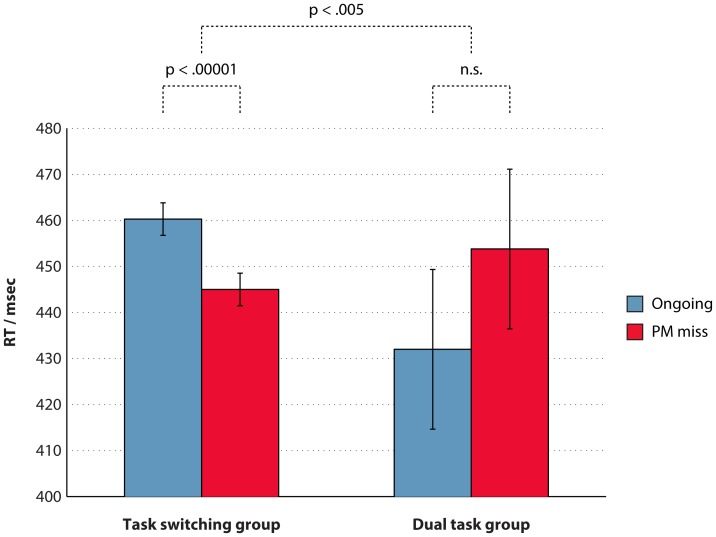
Mean response times for ongoing and PM miss trials in the two groups. The task switching group shows a significant intention superiority effect (i.e. faster responses for PM miss than ongoing trials) but there is no significant difference in the dual task group. Error bars indicate 95% confidence intervals for the within-subjects comparison between the two conditions for each group, using Loftus and Masson’s [49] method.

Table 1 shows a summary of results. In the dual task group, any target trial in which the PM button was pressed was counted as a PM hit. In the task switching group only the first button pressed on each trial was considered in order to calculate PM accuracy. Thus, the definition of a PM hit differed between the two groups in accordance with the instructions that they were given. Results from the task switching group conformed with the predictions from the model. PM miss RTs were faster than ongoing RTs, i.e. there was an intention superiority effect (F(1,19) = 41, p<.001, η^2^_p = _.69). As in Experiment 1, Wilcoxon tests were used to compare unbiased estimates of CV between the conditions. Ongoing trials had significantly greater CV than both PM hits (p<.001) and PM misses (p = .04). Thus, the task switching group replicated the findings of Experiment 1, further confirming the predictions of the model. However, if anything, the dual task group showed a “intention inferiority effect”, i.e. slower RTs for PM miss than ongoing trials (F(1,19) = 3.5, p = .08, η^2^_p = _.16). One participant in the dual task group showed an “intention inferiority effect” of 220 ms, more than 8 standard deviations from the rest of the group, and was therefore excluded as an outlier in this analysis. Even with this participant included in the analysis, the RT difference between ongoing and PM miss trials was still not significant (p = .08). Direct comparison indicated a significant difference between the intention superiority effects of the two groups (F(1,38) = 9.7, p = .004, η^2^_p = _.21). This difference between the groups is illustrated in Figure 8. Furthermore, in the dual task group the CV findings were reversed: ongoing CV was significantly *lower* than both PM hit CV (p = .001) and PM miss CV (p = .01). Mann-Whitney U tests showed a significant difference between the two groups in both CV differences (p<.002). Thus, although the performance of the task switching group was similar to the performance of the model (which implements a task switching version of the task), participants performing a noncompetitive version of the task did not perform in the manner predicted by the model. Analysis of individual differences in PM hit rates showed that the intention superiority effect was not significantly correlated with the hit rate in either group (task switching: r = .11, p = .64; dual task: r = −.23, p = .35). Comparison of these correlation coefficients with Experiment 1 showed that this correlation was significantly lower than the previous experiment in the dual task group (p = .004) and marginally-significantly so in the task switching group (p = .07).

**Table 1:** Results of Experiment 2, presented separately for the task switching and dual task groups, along with statistical comparisons between the two groups.

	Mean		Comparison
	*Task switching*	*Dual task*	
PM hit rate	31%	71%	t(38) = 7.8, p<.001
PM hit RT	601 msec	690 msec	t(38) = 5.2, p<.001
PM hit CV	0.13	0.29	Mann-Whitney U test, p<.001
PM false alarmrate	0.47%	0.99%	t(38) = 2.2, p = .03
Ongoing RT	460 msec	432 msec	t(38) = 1.5, p = .15
Ongoing accuracy	93%	91%	t(38) = 1.1, p = .30
Ongoing CV	0.18	0.19	Mann-Whitney U test, p = .50
PM miss RT	445 msec	454 msec	t(38) = 0.4, p = .72
PM miss CV	0.17	0.23	Mann-Whitney U, p = .003

## Discussion

This experiment arose from the consideration that our model depends on competition between the ongoing and PM pathways in order to produce three effects: 1) intention superiority effect; 2) greater CV of ongoing than PM miss RT distribution; 3) greater CV of ongoing than PM hit RT distribution. If these phenomena were to be found regardless of whether the task was performed in a competitive or noncompetitive manner, this would cast doubt on the generalizability of the model’s predictions. However, we found that the intention superiority effect was eliminated and the CV effects were reversed when participants performed a noncompetitive version of the task. This provides further evidence for the importance of competition for the findings simulated by the model. As discussed in the Introduction, it is likely that some of the phenomena reported in the PM literature (e.g. intention superiority effect, intention maintenance cost) result from multiple causes, only some of which are simulated in the model. We therefore do not propose that these phenomena should only ever be seen in situations simulated by the model. However, the finding that the three phenomena investigated in this experiment were either abolished or reversed in conditions dissimilar to those simulated by the model lends further support to its utility in understanding at least some of the origins of empirical phenomena reported in the PM literature. These findings also underline the importance of considering the generalizability of phenomena across diverse empirical approaches for the study of PM.

Unlike Experiment 1, the intention superiority effect was not correlated with the PM hit rate in either group in this experiment. This is perhaps surprising, at least in the task switching group. How might this discrepancy be explained? One possibility is that this result simply reflects noise, seeing as the direct comparison between Experiment 1 and the task switching group was only marginally significant. However, an alternative account might be as follows. In Experiment 1, the stimulus on each trial remained on the screen until the response was made. Thus participants could choose to delay their ongoing response as long as they liked if they wanted to ensure that they did not miss PM targets. In this case, considerable variance in PM hit rates might be explained by individual differences in PM monitoring, associated with perceived priority of PM versus ongoing task demands or the conscientiousness with which participants performed the task. The simulations above showed that manipulating PM hit rates by adjusting the monitoring level led to a correlation between PM hit rates and the intention superiority effect. However, in Experiment 2, stimuli were presented for a fixed period of 650 msec, so that stimulus presentation times could be equalized between the two groups and in order to avoid ceiling effects in PM performance. Correspondingly, ongoing RTs were considerably shorter in this experiment, and PM hit rates were lower in the task switching group (comparable to the instructions for Experiment 1). Thus, in the present experiment, relatively little variance in PM hit rates might be explained by individual differences in PM monitoring, seeing as participants could not choose to delay their ongoing responses indefinitely in order to ensure accurate PM responding. In this experiment, variance in PM hit rates might be more readily explained in terms of intrinsic capacity limitations, which might not be expected to correlate with the intention superiority effect. This interpretation might underlie the difference between Experiment 1 and Experiment 2, although in the absence of direct empirical evidence it remains a speculative suggestion at present.

### Simulation of Intention Encoding

The simulations considered above involve a model whereby the identity of target stimuli (i.e. repeated letters) is hard-coded into the connection weights. In this section, we consider how this model might be extended to simulate a situation in which the identity of PM targets can be dynamically updated, i.e. where the model can encode the identity of new target stimuli from one trial to the next. The situation we consider is similar to the paradigm investigated in an fMRI study reported by Gilbert et al. [30]. In this study, participants viewed a series of stimuli as part of an ongoing task, some of which were surrounded by a coloured border. When this occurred, participants memorized the stimulus so that they could make a PM response if they encountered that stimulus on a subsequent trial, rather than perform the ongoing task (see [31] for a related approach). Gilbert et al. [30] investigated the correlation between patterns of brain activity on the trial when a cue was encoded versus the trial when it was repeated (i.e. PM target trial). They found that this correlation was higher for PM hits than PM misses. In other words, successful PM performance was associated with enhanced correlation between encoding- and retrieval-related brain activity. Here, we investigate whether a model that incorporates both PM encoding and PM detection produces a similar pattern of results.

For this simulation, we only considered a single PM target stimulus, rather than the multiple PM target stimuli in the earlier simulations (“A a”, “B B”, etc.). Thus we removed two of the target detection units so that there was only a single target detector. Furthermore we removed the hard-wired connections from stimulus input units to the target detecting unit. Apart from this, the architecture and connection weights of the model were left unchanged. In order to encode a PM target, a stimulus was presented at the stimulus input units (i.e. two units had their activation levels set to 1). Furthermore, noise was applied to each stimulus input unit to simulate variability between perceptual processing and contextual factors from one trial to the next. This was achieved by adding a random number drawn from a Gaussian distribution (mean: 0, standard deviation: 0.15) to each stimulus input unit. Note that activation of the stimulus input units could just as well represent internal simulation of a target stimulus (e.g. visual imagery of that stimulus, which is known to yield patterns of brain activity in low-level visual cortex akin to actually viewing a particular stimulus) rather than perception of that stimulus (see [30], p. 103, for further discussion of this point). The target detector unit also had its activation level set to 1. Following this, Hebbian learning was applied between each of the stimulus input units and the target detection unit, i.e. the connection weight from each stimulus input unit to the target detection unit was set to the product of the two activation levels (seeing as this product was not scaled, this corresponds to a learning rate of 1). The model was then run as before, with 100 repetitions of each possible stimulus apart from the PM target, which was presented an equivalent number of times as all of the other stimuli summed together. During this testing phase, the stimulus input units selected on each trial had random noise added in the same manner as the encoding phase, to simulate variability in perceptual processing and contextual factors. This procedure was repeated 1000 times, with a randomly selected target stimulus each time, in order to assess the model’s performance. In order to assess encoding-retrieval similarity of representations, the correlation coefficient was obtained between activation levels of the stimulus input units on each stimulus encoding trial and its associated target trials.

Three features of the model’s behaviour are of note. First, the model performs the task adequately (PM hit rate: 62%; PM false alarm rate: 0.7%; ongoing accuracy: 99%). Second, the model continues to simulate the intention superiority effect (ongoing RT: 54.0 cycles; PM miss RT: 47.2 cycles), and also the CV for ongoing trials (0.55) was greater than the CV for PM hits (0.39) or PM misses (0.45). Thus, the model consistently simulates the phenomena described in the earlier simulations. Third, as in the fMRI data reported by Gilbert et al. [30] the similarity (i.e. correlation coefficient) between encoding-related and target-related patterns of activation was higher for PM hits than PM misses (hits: r = .887; miss: r = .869). The computational explanation for this is straightforward: as a simple byproduct of the Hebbian learning algorithm, the closer the match between the original presentation of a stimulus and its subsequent presentations, the greater the activation sent to the PM response node. Thus the present modelling framework suggests a simple manner in which the encoding of PM targets could be simulated, and as a natural consequence of this approach it reproduces an effect observed in a neuroimaging investigation of PM. This mirrors a behavioural effect whereby PM performance is boosted when the PM target episode matches the encoding episode more closely, including contextual factors such as the typeface in which items are presented or the room in which participants are seated [32]; [33]; [34].

The simulation above describes a simple method whereby the model can activate a new intention. But what about deactivating intentions? In order to investigate this, an additional simulation was conducted whereby instead of zeroing the connection weights after an intention was no longer relevant, the monitoring level was set to zero and a post-PM block was run. This simulates the persistence of a prior memory trace, even when it is no longer relevant to the task. All other features of the model’s operation were left unchanged. In this simulation, PM “hits” (i.e. errors of commission) occurred on 9.4% of trials (versus a false alarm rate to nontarget items of 0.1%). Ongoing RTs to previous targets were slower (55.8 cycles) than non-target stimuli (53.1 cycles). When connection weights between stimulus input units and the target detection unit were additionally reduced by 50% in the post-PM block, to simulate a decay of the representations of previous targets, this strongly reduced the likelihood of errors of commission (0.2% of trials, versus 0.05% false alarm rate). Nevertheless, RTs to previous targets remained slower (54.7 cycles) than non-target stimuli (53.0 cycles). Thus response competition, caused by associative links that were not strong enough to reliably yield PM responses, nevertheless slowed RTs. This simulates the pattern of results seen in recent studies of intention deactivation, where previous targets slow ongoing responses, in the context of occasional errors of commission [27]; [35]. Note that this slowing could also be considered an “intention inferiority effect”, underlining the importance of the model’s ability to simulate both speeded PM miss RTs (versus ongoing RTs) and slowed PM miss RTs in different circumstances.

## General Discussion

In this article we have presented a computational model of event-based PM with the following core features: 1) competing, interactive pathways governing ongoing and PM responding; 2) two mechanisms underlying PM responding: direct triggering of PM responses by spreading activation from input representations, and assistance from a top-down control mechanism; 3) a graded continuum between controlled top-down monitoring for PM targets versus pure bottom-up triggering, rather than an all-or-nothing mechanism. The model fits a wide body of empirical results: slowing of ongoing responses and increased PM accuracy as a result of top-down monitoring for PM targets; correlation between ongoing RT and PM accuracy in some circumstances but not others; and the intention superiority effect (i.e. faster PM miss than ongoing RT). The model also made novel predictions that were confirmed in two empirical studies. PM accuracy was reduced (and ongoing RT increased) when visual stimuli were degraded. Furthermore, the model predicted that ongoing trials should differ from PM miss and PM hit trials in the shape of RT distributions; analogous effects were found in the empirical data. Finally, the model predicted that these RT distribution effects and the intention superiority effect should be modulated by the use of experimental paradigms in which PM responses either accompany or replace ongoing responses. This prediction was also confirmed empirically.

Of course, the model does not provide an account of all empirical phenomena that have been reported in the PM literature; nor, for those phenomena it does simulate, does it necessarily provide an exhaustive account. In particular, the simulations reported above rely heavily on competitive interactions between ongoing and PM pathways. Yet some of the phenomena simulated by the model, including the intention superiority effect and the intention maintenance cost, have been reported in noncompetitive situations where PM responses accompany rather than replace ongoing responses [3]; [6]. This suggests that the model simulates a subset of the cognitive processes contributing to PM performance, and that extra principles must also be considered to account for PM performance across all experimental paradigms. We have already considered in the Introduction some of the alternative, non-exclusive accounts that have been considered for the intention superiority effect. We also note that a variety of different paradigms have been used to investigate the intention superiority effect, often involving memory for script based activities (e.g. [7]) which may involve rather different mechanisms. Regarding the intention maintenance cost, other phenomena that might be considered are response-threshold shifts [36] and additional performance costs associated with monitoring/checking the environment for PM cues that need not apply on ongoing-only trials [3]; [37], alongside the model’s simulation of greater response competition engendered by PM conditions. Furthermore, PM targets were presented relatively frequently (8% of trials) in the empirical work presented here. It is unclear how well the model’s predictions would generalize to paradigms with less frequent target presentation. The present work suggests that processes underlying PM performance can be multifaceted, depending on the precise nature of the experimental task. One corollary of this conclusion is that different processes might well account for identical patterns (e.g. intention superiority effect) across different PM tasks.

It should also be noted that in the present work the model parameters were set by hand, and we have not attempted to systematically explore the parameter space. It is therefore possible that the model might produce alternative patterns of behaviour if different parameter settings were used. Seeing as there are multiple free parameters in the model, it is a complex question how to set upper and lower bounds for those parameters and then explore the multi-dimensional parameter space in a computationally tractable manner (see [38] for discussion). Here, rather than attempting such an enterprise, we have taken the approach of validating the model via the generation and verification of novel predictions, along with showing that it can reproduce previously-reported patterns of results.

Although these considerations make it clear that the model does not provide an exhaustive account of phenomena associated with PM, Experiment 2 showed that some of these phenomena were significantly modulated by subtle changes in the experimental paradigm used for assessing PM, as predicted by the model. This underlines the importance of explicit computational accounts that can help to link specific experimental paradigms to underlying principles. Given the clear differences between the task switching and dual task groups tested in Experiment 2, it seems unlikely that a single computational explanation will account for behavioural data across all of the diverse experimental paradigms that have been used to test PM. However, the successful novel predictions made by the model demonstrate its utility for understanding underlying processes involved in at least some PM paradigms. It is an interesting question how far everyday PM situations should be considered in terms of the task switching versus dual task operationalizations examined in Experiment 2. While in many everyday PM tasks the intended behaviour can be produced in addition to ongoing activities, other situations require the intended behaviour to replace the ongoing activity. These situations include any circumstance in which the intended and ongoing behaviours are mutually incompatible, for example stopping at a shop to buy milk on the way home, instead of continuing one’s journey.

The finding in the model and in participants performing a competitive version of the task that PM hit trials had a lower coefficient of variation (CV) than ongoing trials is of particular theoretical interest. Previous studies have suggested that conditions involving executive function, or controlled processing, lead to an increase in CV, relative to conditions involving more automatic processing. For example, Segalowitz and Segalowitz [24] showed that both RT and CV increased in a second-language lexical decision task for participants with relatively little practice. Additionally, Segalowitz et al. [25] showed, in a task switching paradigm [39], that trials following a switch of task had slower RTs and increased CV. By contrast, at least in the competitive version of the task, PM hit trials (which may be thought to involve control processes such as inhibition of the ongoing response, and monitoring for target events) had slower RTs but reduced CV, in comparison with ongoing trials. Thus, competitive PM paradigms seem to constitute an exception to the rule that tasks involving relatively controlled processing lead to increased CV. This can be explained by the hypothesis that on those trials where participants have difficulty producing a PM response (e.g. due to noise), instead of producing a very slow response, they may actually produce a PM miss response instead. Such trials will therefore not be included as a PM hit, leading to a narrowing of the PM hit distribution. Consistent with this explanation, PM hit trials had increased CV, relative to ongoing trials, in the dual task condition.

### Comparison with Theoretical Accounts of PM

We believe that the present model corresponds most readily with the multiprocess framework put forward by McDaniel and Einstein [13]. Consistent with this account, PM responses in the model can arise either from direct environmental triggering, or as a result of the influence from top-down monitoring. Even when this top-down monitoring system was switched off (i.e. set to zero), the model still made accurate PM responses on a small proportion of trials. Thus, bottom-up triggering was sufficient to enable PM responding on at least some trials, consistent with Scullin et al.’s [11] suggestion that certain circumstances can permit appropriate PM responding in the absence of strategic monitoring.

One of the ways in which the model is currently somewhat limited is in the use of localist stimulus representations, with each unit representing both the identity and the location of a stimulus. A more sophisticated model might make use of distributed representations of different stimulus features. In this case, attentional biases towards stimulus features that are relevant for the ongoing task might have the effect of reducing activation related to features that are irrelevant to the ongoing task. This might help to capture the contrast between bottom-up triggering of PM responses by ‘focal’ versus ‘non-focal’ PM cues [13], where the feature that defines PM targets either overlaps or fails to overlap with an ongoing-task-relevant stimulus feature.

In contrast with the multiprocess framework, Smith [3] has argued that strategic monitoring is always required for PM responding. This framework has been elaborated in a multinomial mathematical model by Smith & Bayen [20] (see also [36]; [40]; [41]). It should be noted that the present modelling framework might be consistent with the hypothesis that PM always requires strategic monitoring, if different parameter settings were adopted. For example, if the connection strengths from the target monitoring unit to the PM response unit were weakened, the model might never be able to make a PM response purely on the basis of bottom-up triggering, and would therefore require top-down control from the monitoring unit. However, we see the model as differing from the framework presented by Smith and Bayen [20] in two fundamental respects. First, in the present model, top-down control is graded along a continuum. By contrast, in Smith and Bayen’s model, top-down control is either engaged or not, in a binary stochastic manner. Second, Smith and Bayen’s model implements a two-stage process: on each trial, the model either is monitoring for a PM target or not. If it is monitoring and a PM target is presented, a PM response will always be made (subject to accurate retrospective memory for the target identity); otherwise a PM response will never be made. There is therefore no direct interaction between PM and ongoing pathways; the model’s preparedness for an ongoing response has no influence on PM performance. However, in the present model, interactive competition between the PM and ongoing pathways plays a fundamental role in its simulation of the intention superiority effect, and the differing RT distributions between ongoing and PM conditions. In the absence of interactive competition between ongoing and PM response pathways, it is difficult to see how the distinction between RT distributions on ongoing and PM trials could be simulated. We therefore suggest that Smith and Bayen’s model can play a helpful role in simulating certain patterns of behaviour, but does not capture phenomena resulting from interactive competition between processing related to ongoing and PM task demands.

### Relationship with Brain Mechanisms Underlying PM

While it may be oversimplistic to identify individual elements of the model with specific brain regions, we wish to point out one potential relationship between the present modelling results and data from neuroimaging. One feature of the monitoring unit in our model is that it is connected equally to all units representing potential PM targets. It might therefore be considered to play a “content-free” role in PM, in the sense that the monitoring unit itself does not represent any specific PM target stimulus, or a specific PM response. These elements are represented elsewhere in the model. The role of the monitoring unit is simply to up-regulate all processing related to PM, without requiring any detailed representation of potential PM targets or responses. This content-free role may be compared with the role of rostrolateral prefrontal cortex (RLPFC) suggested in a functional MRI study by Gilbert [42]. Previous neuroimaging investigations of PM have consistently reported increased activation in RLPFC, corresponding to lateral aspects of Brodmann Area 10, when participants anticipate and/or encounter PM targets [43]; [44]; [45]; [46]. Gilbert [42] showed, in a modified PM paradigm, that the content of delayed intentions could be decoded from patterns of brain activity in medial frontal and posterior brain regions. However, although RLPFC showed strong activity while participants maintained delayed intentions, the content of those intentions could not be decoded from RLPFC itself. Furthermore, RLPFC increased its functional coupling with intention-representing brain regions while intentions were stored. In this respect, the role of RLPFC in PM might be considered to be analogous to the monitoring unit in the present study: interacting with representations stored elsewhere, so that appropriate targets may be detected and responses produced, rather than itself representing specific PM cues and responses.

The finding in Gilbert [42] that functional coupling between RLPFC and intention-representing brain regions was increased during intention storage would be trivially simple to simulate in the present modelling framework. If activation of the monitoring unit were to fluctuate over time during intention storage (cf. [9]), this would lead to a correlation between activation in the monitoring unit and the target detection units to which it is directly connected. By contrast there would be no correlation between activation in the monitoring unit and target detection units if the monitoring level were set to zero.

The present simulations also captured an additional phenomenon consistent with neuroimaging data, namely a greater correlation between encoding- and retrieval-related activity for hits than misses [30]. Note that in both the empirical and the computational data a similar analysis was conducted: calculation of the correlation coefficient between two vectors of activation levels, with each vector representing a specific trial or condition. This approach, in the context of neuroimaging, has previously been referred to as ‘representational similarity analysis’ [47]. However, the same technique is just as applicable to the type of model investigated here: a similar conclusion is drawn regardless of whether the analysis is conducted over a vector of parameter estimates across a set of voxels [30] or across a vector of activation levels across a set of processing units (present simulations). Connectionist computational models can be difficult to connect with results from neuroimaging, seeing as such models do not always make anatomical predictions suitable for testing with such techniques [48]. However, representational similarity analysis provides an approach that allows us to bring together behaviour, computational modelling, and functional neuroimaging within a single framework.

### Conclusions

The present results lend support to the hypothesis that event-based PM depends on the interplay between bottom-up triggering of appropriate responses by environmental cues, and top-down monitoring. The results also suggest that an important determinant of behaviour in at least some PM paradigms may be interactive competition between processing pathways supporting ongoing versus PM responses. We hope that this relatively simple modelling framework may serve as a bridge to link cognitive-level theories of the processes underlying PM with neuroscientific investigations of its underlying brain mechanisms.

## Supporting Information

Table S1 **Standard parameter settings.**
(DOCX)
