# Supplementary material for: Automaticity and Control in Prospective Memory: A Computational Model
Source: PLoS One. 2013 Mar 28;8(3):e59852. doi: 10.1371/journal.pone.0059852 (PMC3610891; doi:10.1371/journal.pone.0059852)
Supplement: Table S1 — Standard parameter settings. (DOCX) [file pone.0059852.s001.docx]

**Table S1.** Standard parameter settings.

| **Parameter** | **Standard setting** |
| --- | --- |
| Weights |  |
| Input to output (ongoing) | 1.5 |
| Input to target detection | 1.0 |
| Target detection to output | 8.0 |
| Lateral inhibition (target detection and output units) | -3 |
| Other |  |
| Bias for target detection units | -2 |
| Noise (standard deviation of noise distribution) | 0.015 |
| Response threshold | 0.5 |
| Maximum unit activation | 1.0 |
| Minimum unit activation | -0.1 |
| Monitoring level | 0.7 |
| Step size | 0.01 |
